# Immunological effects of subcutaneous and sublingual immunotherapy in house dust mite-allergic adults: a nine-month prospective pilot study

**DOI:** 10.1038/s41598-026-56260-8

**Published:** 2026-06-09

**Authors:** Sara I. Taha, Aya Tollah Shaban, Afaf Abdelalim Mostafa, Sara S. Ghonaim, Lamyaa Salem

**Affiliations:** 1 Department of Clinical Pathology, Faculty of Medicine, Ain-Shams University, Abbasia, Cairo, 11591 Egypt; 2 Department of Medical Laboratory Technology, Faculty of Applied Health Science, El-Galala University, Suez, Egypt; 3 Department of Internal Medicine, Allergy and Clinical Immunology, Faculty of Medicine, Ain Shams University, Cairo, Egypt

**Keywords:** House dust mite, Allergen immunotherapy, IgG4, Regulatory T cells, Asthma, Allergic rhinitis, Quality of life, Biomarkers, Diseases, Immunology, Medical research

## Abstract

House dust mite (HDM) allergy contributes to allergic rhinitis and asthma worldwide. Allergen-specific immunotherapy (AIT) is the only disease-modifying treatment, with immunoglobulin G4 (IgG4) and regulatory T cells (Tregs) mediating immune tolerance. Comparative immunological effects of subcutaneous (SCIT) versus sublingual (SLIT) therapy remain underexplored. To evaluate immunological changes induced by SCIT and SLIT and their association with clinical improvement in HDM-allergic patients. In this prospective cohort, 43 adults with HDM-sensitized allergic patients received SCIT (n = 22) or SLIT (n = 21) for nine months. Clinical outcomes were assessed using the Asthma Control Test (ACT) and Rhinitis Control Assessment Test (RCAT). Serum IgG4 and total IgE were measured by ELISA and electrochemiluminescence, respectively, and CD4⁺CD25⁺FoxP3⁺ Tregs were analyzed by flow cytometry. Responders were defined as patients achieving ≥ 20% improvement in ACT or RCAT. Wilcoxon signed-rank, Mann–Whitney U, and Spearman correlation tests were used. AIT increased IgG4 (320 → 920 ng/mL; *p* < 0.001) and Tregs (3.4% → 6.3%; *p* < 0.001), with a non-significant decrease in IgE. Responders had higher IgG4 and Tregs and lower IgE than non-responders. SCIT elicited higher IgG4 levels (median 1035 vs 705 ng/mL) and a trend toward greater Treg expansion compared with SLIT, although clinical improvement was similar between groups. IgG4 correlated with ACT (*p* < 0.001) and RCAT (p = 0.002), and Tregs correlated positively with IgG4 (p = 0.003) and inversely with IgE (p = 0.010). Both SCIT and SLIT improve clinical outcomes in HDM-allergic patients via total IgG4 elevation and Treg expansion. SCIT may induce stronger systemic immunological responses, supporting the use of these biomarkers for early monitoring and personalized therapy. These findings should be interpreted within the context of a pilot study with a relatively small sample size.

## Introduction

Respiratory allergic diseases, including allergic rhinitis and allergic asthma, represent a major global health burden affecting hundreds of millions of individuals worldwide. These disorders are characterized by immunoglobulin E (IgE)-mediated hypersensitivity responses to environmental allergens, leading to chronic airway inflammation and recurrent respiratory symptoms^1,2^. Among inhalant allergens, house dust mites (HDM) are considered one of the most common causes of perennial allergic sensitization and are strongly associated with both allergic rhinitis and asthma severity^3–5^.

Allergen-specific immunotherapy (AIT) remains the only disease-modifying treatment capable of altering the natural course of allergic diseases. Unlike conventional pharmacotherapy, which mainly controls symptoms, AIT induces long-term immune tolerance to specific allergens^6,7^. The two most widely used routes of administration are subcutaneous immunotherapy (SCIT) and sublingual immunotherapy (SLIT). Both approaches have demonstrated efficacy in reducing symptoms and medication use in allergic patients; however, differences in their immunological mechanisms and clinical responses remain an area of active investigation^8^. Importantly, beyond symptom control, AIT has been associated with significant improvements in patients’ quality of life, as better disease control translates into enhanced daily functioning, sleep quality, and overall well-being^9^.

The therapeutic effects of AIT are mediated through complex immunological mechanisms involving immune deviation and induction of peripheral tolerance^8,10^. One of the hallmarks of successful immunotherapy is the induction of immunoglobulin G4 (IgG4) antibodies^11^. IgG4 is considered a “blocking antibody” that competes with IgE for allergen binding, thereby preventing allergen-IgE interactions and subsequent mast cell and basophil activation^12^. Increased levels of total IgG4 during immunotherapy have been associated with clinical improvement and are widely used as an immunological marker of treatment response^11^.

In addition to humoral changes, cellular immune regulation plays a critical role in the development of allergen tolerance. Regulatory T cells (Tregs), particularly CD4⁺CD25⁺FoxP3⁺ T cells, are central mediators of immune tolerance induced by AIT. These cells suppress allergen-specific T helper 2 (Th2) responses and promote the secretion of anti-inflammatory cytokines such as interleukin-10 (IL-10) and transforming growth factor-β (TGF-β). Through these mechanisms, Tregs contribute to the down-regulation of IgE production and the promotion of IgG4 synthesis by B cells^13–15^.

Despite increasing evidence supporting the role of these biomarkers, comparative studies evaluating the immunological changes induced by SCIT versus SLIT in HDM-allergic patients remain limited. Understanding these immunological differences may help optimize patient selection and monitoring strategies for allergen immunotherapy.

Therefore, the present study was designed to investigate the immunological effects of HDM immunotherapy by measuring serum total IgG4, total IgE, and Tregs expressing FoxP3 before and after treatment in patients receiving either SCIT or SLIT. Furthermore, the study aimed to evaluate whether these immunological parameters could serve as biomarkers for comparing the efficacy of the two immunotherapy modalities.

## Methods

### Study design and setting

This prospective cohort pilot study was conducted on 43 adult patients attending the outpatient clinics of the Internal Medicine and Allergy and Immunology Departments at Faculty of Medicine, Ain Shams University, Cairo, Egypt. Patients were consecutively recruited and followed to evaluate immunological changes associated with AIT.

### Ethical considerations

The study protocol was reviewed and approved by the Institutional Research Ethics Committee of the Faculty of Medicine, Ain Shams University (approval number FMASU MS 665/2023). All procedures were conducted in accordance with the ethical principles outlined in the Declaration of Helsinki of the World Medical Association. Written informed consent was obtained from all participants prior to enrollment in the study.

### Study population

Eligible participants were adult patients aged between 18 and 60 years who were diagnosed with bronchial asthma and allergic rhinitis. The diagnosis of bronchial asthma was established according to the recommendations of the Global Initiative for Asthma (GINA) Guidelines^16^, while allergic rhinitis was diagnosed based on the criteria of the Allergic Rhinitis and its Impact on Asthma (ARIA) Guidelines^17^. In addition, all participants demonstrated sensitization to HDM allergens as confirmed by a positive skin prick test.

Patients were excluded if they had received previous or ongoing AIT, as this could affect immunological baseline measurements. Pregnant or lactating women were also excluded due to safety considerations related to immunotherapy administration. Patients with dermographism were excluded because this condition may interfere with the interpretation of skin prick test results. Individuals with severe or uncontrolled bronchial asthma were excluded because allergen immunotherapy may increase the risk of systemic reactions in such cases. Furthermore, patients with autoimmune diseases, malignancies, or significant chronic medical comorbidities were excluded to avoid confounding effects on immune responses and immunoglobulin levels.

### Clinical evaluation

All participants underwent a comprehensive clinical evaluation that included detailed medical history taking and physical examination. Asthma control was assessed using the validated Asthma Control Test (ACT), which consists of five patient-reported questions evaluating asthma symptoms, rescue medication use, and overall disease control during the preceding four weeks. ACT scores range from 5 to 25, with higher scores indicating better asthma control^18^. Allergic rhinitis symptom control was assessed using the Rhinitis Control Assessment Test (RCAT), a validated six-item questionnaire designed to evaluate nasal symptoms, sleep disturbance, and activity limitation related to rhinitis over the previous week. Higher scores indicate better symptom control^19^.

Patients were classified as responders or non-responders based on improvement in clinical scores after nine months of immunotherapy. Responders were defined as patients demonstrating ≥ 20% improvement in ACT or RCAT scores compared with baseline, whereas those with < 20% improvement were classified as non-responders. This threshold was chosen as an exploratory cutoff based and was intended to provide preliminary insights into the immunological correlations of treatment success.

### Skin prick testing

Skin prick testing was performed to confirm sensitization to HDM allergens. Standardized allergen extracts were applied to the volar surface of the forearm, and the skin was gently punctured using sterile disposable lancets. Histamine was used as a positive control and normal saline as a negative control. The wheal-and-flare reaction was measured after 15 min, and a wheal diameter of at least 3 mm greater than that of the negative control was considered a positive result indicating sensitization^20^.

### Sample collection

Peripheral venous blood samples were obtained from all participants before initiation of AIT and after nine months of AIT. Blood samples were divided into ethylenediaminetetraacetic acid (EDTA) tubes for complete blood count (CBC) analysis and flow cytometry, and plain tubes for serum separation. Serum samples were obtained after centrifugation at 3000 rpm for 20 min and stored at − 80°C until analysis of IgG4.

### Laboratory investigations

CBC analysis was performed using the automated hematology analyzer Sysmex XN-1000 manufactured by Sysmex Corporation, Kobe, Japan, according to the manufacturer’s instructions.

Serum total IgE concentrations were measured using an electrochemiluminescence immunoassay (ECLIA) on the Cobas e 411 analyzer developed by Roche Diagnostics, Mannheim, Germany. Results were expressed in international units per milliliter (IU/mL).

Serum total IgG4 levels were determined using a human enzyme-linked immunosorbent assay (ELISA) kit (Cat. No. EA0088HU) supplied by Bioassay Technology Laboratory, Shanghai, China. The assay was performed according to the manufacturer’s instructions. Optical density was measured at 450 nm using a microplate reader, and IgG4 concentrations were calculated from a standard calibration curve generated during the assay.

### Flow cytometric analysis of Tregs

Tregs expressing FoxP3 were analyzed using a Navios Flow Cytometer (six-color system) manufactured by Beckman Coulter, USA. Peripheral blood samples were stained with fluorochrome-conjugated monoclonal antibodies including anti-CD4-FITC (Cat. No. A07750; Clone: 13B8.2), anti-CD25-PE (Cat. No. IM0479U; Clone: B1.49.9), and anti-FoxP3-AF647, (Cat. No. B30650; Clone: 259D), all supplied by Beckman Coulter, Marseille, France.

The gating strategy involved initial identification of lymphocytes on a forward scatter (FSC) versus side scatter (SSC) plot. CD4⁺ T helper lymphocytes were then gated within the lymphocyte population. Subsequently, CD25 expression was evaluated within the CD4⁺ T cell subset. Intracellular FoxP3 expression was finally analyzed within the CD4⁺CD25⁺ population to identify Tregs. Results were expressed as the percentage of CD4⁺CD25⁺FoxP3⁺ cells among total CD4⁺ T lymphocytes.

#### Statistical analysis

Data were analyzed using SPSS version 28 (IBM Corp., Armonk, NY, USA). Continuous variables were expressed as median and interquartile range (IQR). Within-group comparisons before and after immunotherapy were performed using the Wilcoxon signed-rank test, while between-group comparisons (SCIT vs SLIT, responders vs non-responders) were assessed using the Mann–Whitney U test. Receiver operating characteristic (ROC) curve analysis was performed to assess the ability of serum IgG4 levels and Treg percentages to discriminate responders from non-responders. The area under the curve (AUC) and optimal cutoff values were calculated. Correlations between immunological and clinical parameters were evaluated using Spearman’s rank correlation coefficient. Categorical variables were compared using the chi-square test. A two-tailed *p* value < 0.05 was considered statistically significant.

## Results

### Baseline characteristics of the study population

A total of 43 patients with HDM-sensitized respiratory allergic disease were included in the study. Twenty-two patients received SCIT and twenty-one patients received SLIT. The median age of participants was comparable between the two groups, and there were no statistically significant differences regarding sex distribution or baseline clinical scores. Additional baseline characteristics, including smoking status, disease duration, comorbidities, and concomitant medication use, were also evaluated and found to be comparable between the SCIT and SLIT groups, with no statistically significant differences observed (*p* > 0.05 for all comparisons). Table 1

**Table 1:** Baseline demographic and clinical characteristics of the patients studied.

Variable	SCIT (n = 22)	SLIT (n = 21)	*p* value
Age (years) median (IQR)	31 (24–39)	30 (23–38)	0.68
Sex (male/female) n & (%)	12 (54%) / 10 (46%)	11 (52%) / 10 (48%)	0.91
Smoking status (smoker/non-smoker) n & (%)	6 (27%) / 16 (73%)	5 (24%) / 16 (76%)	0.82
Disease duration (years) median (IQR)	5 (3–8)	4 (2–7)	0.57
Allergic rhinitis only/Rhinitis + asthma n & (%)	9 (41%) / 13 (59%)	8 (38%) / 13 (62%)	0.84
Comorbidities (yes/no) n & (%)	7 (32%) / 15 (68%)	6 (29%) / 15 (71%)	0.79
Concomitant medications (yes/no) n & (%)	18 (82%) / 4 (18%)	17 (81%) / 4 (19%)	0.92
ACT score median (IQR)	16 (14–18)	16 (15–18)	0.74
RCAT score median (IQR)	18 (16–20)	19 (17–21)	0.62

ACT, Asthma Control Test; RCAT, Rhinitis Control Assessment Test.

### Changes in immunological biomarkers after immunotherapy

**Fig. 1:**
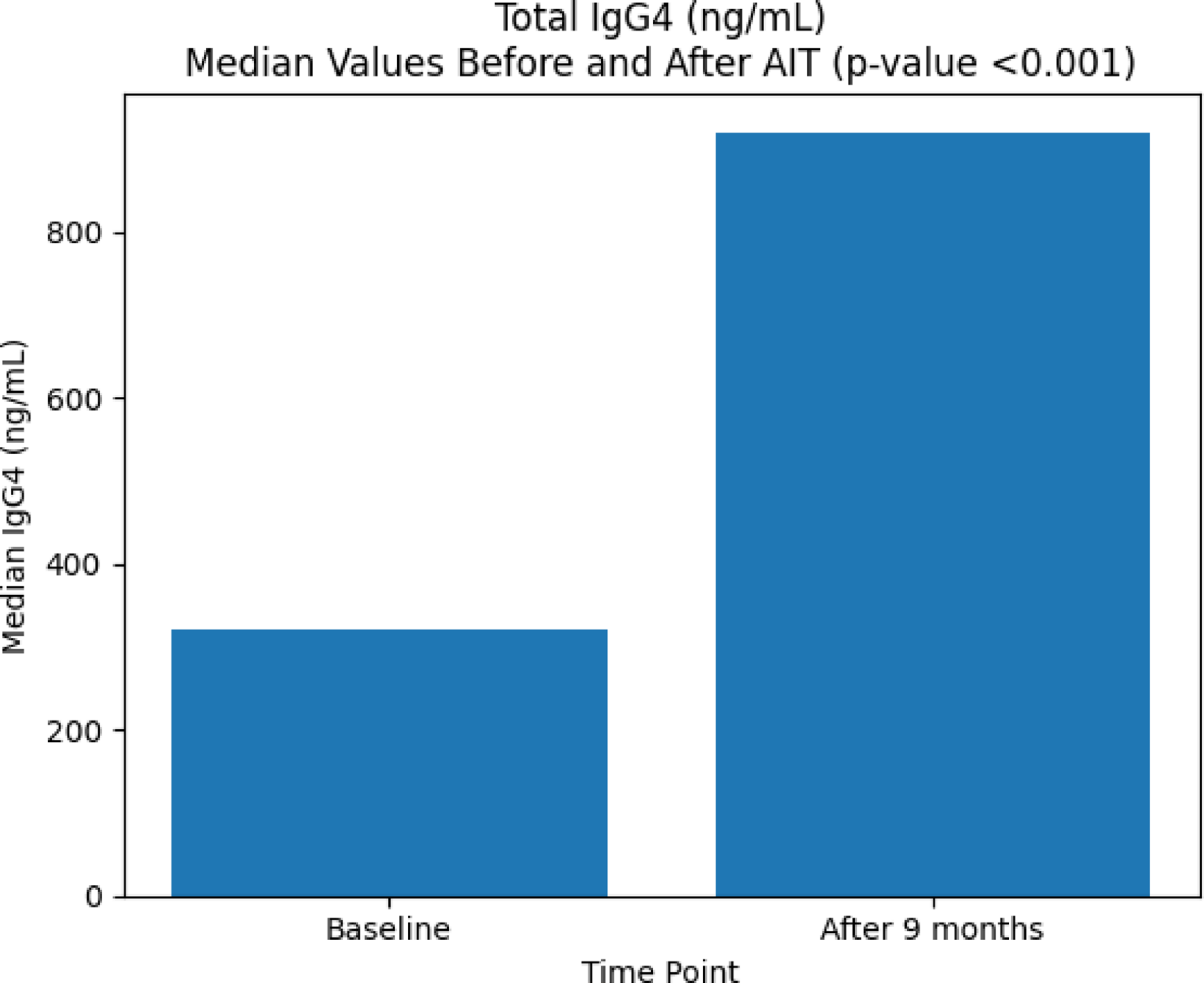
Comparison of total IgG4 before and after AIT.

Evaluation of humoral immune responses demonstrated a marked increase in serum IgG4 levels after nine months of AIT. The median IgG4 concentration increased significantly from 320 (IQR: 150–600) at baseline to 920 ng/mL (IQR: 540–1400) after treatment (*p* < 0.001). In contrast, total IgE levels showed a mild reduction that did not reach statistical significance (p = 0.112). Table 2 and Fig. 1

**Table 2:** Changes in immunological parameters before and after AIT.

Parameter	Baseline median (IQR)	After 9 months median (IQR)	p-value
Total IgE (IU/mL)	240 (120–850)	210 (110–780)	0.112
Total IgG4 (ng/mL)	320 (150–600)	920 (540–1400)	**< 0.001**
CD4⁺ T cells (%)	38 (34–43)	39 (35–44)	0.210
Regulatory T cells (%)	3.4 (2.5–4.2)	6.3 (5.1–7.4)	**< 0.001**

Bold *p* value is significant.

**Fig. 2:**
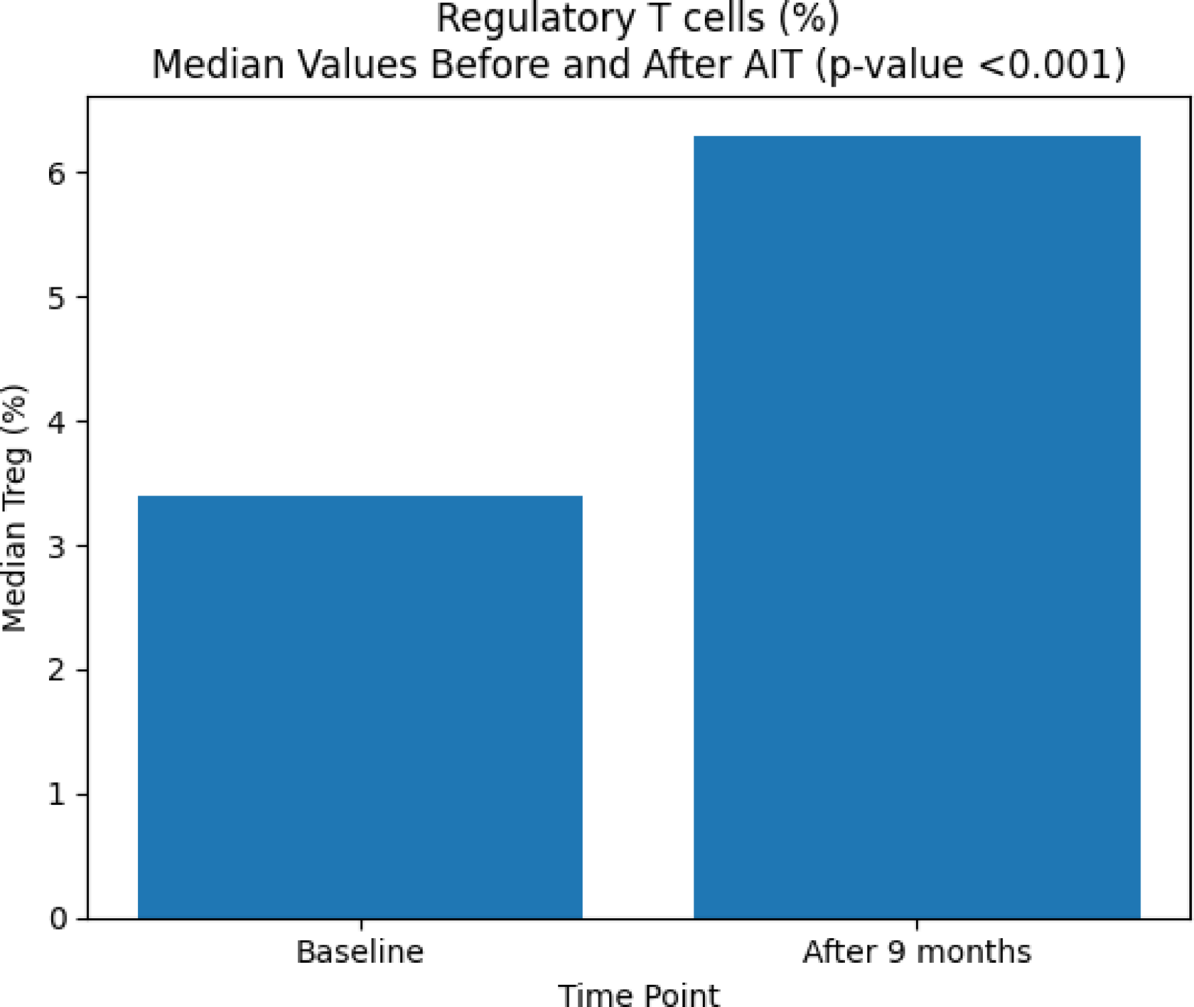
Comparison of regulatory T cells % before and after AIT.

Regarding cellular immune parameters, the percentage of CD4⁺ T helper lymphocytes remained relatively stable during the study period, with no significant change observed after therapy. However, a significant expansion of Tregs was detected following treatment. The proportion of CD4⁺CD25⁺FoxP3⁺ T cells increased from a median of 3.4% (IQR: 2.5–4.2) before therapy to 6.3% (IQR: 5.1–7.4) after nine months of AIT (*p* < 0.001). Table 2 and Fig. 2

### Comparison between SCIT and SLIT after therapy

After nine months of treatment, patients receiving SCIT exhibited higher serum IgG4 levels compared with those receiving SLIT, and the difference was statistically significant (p = 0.005). In contrast, no significant differences were observed between the two groups regarding total IgE concentrations.

Evaluation of cellular immune responses revealed that the percentage of CD4⁺ T helper cells was comparable between both groups. However, the proportion of T regs was higher in the SCIT group compared with the SLIT group, suggesting a stronger induction of immune tolerance with subcutaneous immunotherapy, although this difference did not reach statistical significance (p = 0.058).

Clinical outcomes assessed using ACT and RCAT scores showed improvement in both groups with no significant difference between SCIT and SLIT recipients. Table 3

**Table 3:** Comparison between SCIT and SLIT groups after 9 months of AIT.

Parameter	SCIT (n = 22) median (IQR)	SLIT (n = 21) median (IQR)	*p* value
Total IgG4 (ng/mL)	1035.0 (750.5–1375.2)	705.5 (450.5–1337.5)	**0.005**
Total IgE (IU/mL)	142 (93.5–829)	155.5 (135–537)	0.835
CD4⁺ T cells (%)	39 (35–44)	38 (34–43)	0.091
Regulatory T cells (%)	6.8 (5.3–7.9)	5.9 (4.7–7.0)	0.058
ACT score	22 (21–24)	20.5 (20–21)	0.089
RCAT score	25 (23–28)	24 (23–26)	0.052

ACT, Asthma Control Test; RCAT, Rhinitis Control Assessment Test.

Bold *p* value is significant.

### Responder vs non-responder comparison

Of all patients, 28 (65%) were classified as responders, and 15 (35%) as non-responders. The distribution of SCIT vs SLIT was comparable between the two groups (p = 0.72). Responders exhibited significantly higher total IgG4 levels (median 1050 ng/mL, IQR 720–1400) compared with non-responders (median 620 ng/mL, IQR 400–910; p = 0.002). Treg percentages were also greater in responders (median 7.0%, IQR 5.5–8.0) than in non-responders (median 4.8%, IQR 3.9–5.5; *p* < 0.001). Total IgE levels were lower in responders (median 145 IU/mL, IQR 110–240) compared with non-responders (median 220 IU/mL, IQR 150–350; p = 0.045). No significant difference was observed in CD4⁺ T-cell percentages between the two groups (p = 0.21). Table 4

**Table 4:** Immunological changes in responders vs non-responders.

Parameter	Responders (n = 28) median (IQR)	Non-responders (n = 15) median (IQR)	*p* value
Total IgG4 (ng/mL)	1050 (720–1400)	620 (400–910)	**0.002**
Total IgE (IU/mL)	145 (110–240)	220 (150–350)	**0.045**
CD4⁺ T cells (%)	39 (35–44)	38 (33–42)	0.21
Regulatory T cells (%)	7.0 (5.5–8.0)	4.8 (3.9–5.5)	**< 0.001**

Bold *p* value is significant.

### Prediction of treatment response

ROC curve analysis was performed to evaluate the ability of serum IgG4 levels and regulatory T-cell percentages to discriminate responders from non-responders after nine months of AIT. Serum IgG4 levels demonstrated good predictive performance for treatment response. The area under the ROC curve (AUC) for IgG4 was 0.82 (95% CI: 0.69–0.94, *p* < 0.001). An optimal cutoff value of 850 ng/mL was identified, yielding a sensitivity of 78% and specificity of 73% for predicting clinical response.

Similarly, Treg percentages showed significant predictive ability. The ROC curve for Tregs demonstrated an AUC of 0.79 (95% CI: 0.65–0.92, p = 0.002). A cutoff value of 6.0% provided a sensitivity of 75% and specificity of 70% for identifying responders.

### Correlations between immunological and clinical parameters

Significant positive correlations were observed between total IgG4 levels and ACT (r = 0.62, *p* < 0.001) and RCAT scores (r = 0.58, p = 0.002).

In addition, Tregs demonstrated a significant positive correlation with IgG4 levels (r = 0.55, p = 0.003). Conversely, a significant inverse correlation was detected between Tregs and total IgE levels (r = − 0.48, p = 0.010).

## Discussion

In this study, AIT in HDM allergic patients induced significant immunological modulation, reflected by increased serum total IgG4 and expansion of Tregs. Previous studies reported that in addition to reducing IgE binding and activation of mast cells and basophils, IgG4 can interact with low-affinity Fcγ receptors, such as FcγRIIb, on B cells and other immune cells, thereby inhibiting IgE-facilitated allergen presentation and downregulating pro-allergic signaling pathways^12,21^. This immunomodulation is supported by recent clinical evidence demonstrating that IgG4 not only increases quantitatively during AIT but also correlates with reduced effector cell responsiveness and clinical improvement in allergic asthma and HDM allergy^22,23^. Our data extend this concept by showing that responders exhibit higher IgG4 than non-responders and that IgG4 correlates with clinical scores, reinforcing its utility as a surrogate marker of therapeutic efficacy. Importantly, in our study, total IgG4 increased despite minimal changes in total IgE levels, suggesting that immunotherapy can alter antibody responses independently of overall IgE dynamics.

Additionally, there was a significant expansion of CD4⁺CD25⁺FoxP3⁺ Tregs after AIT highlights the central role of peripheral T-cell tolerance after AIT. Tregs contribute to immune tolerance by suppressing Th2 responses and promoting regulatory cytokines such as IL-10 and TGF-β, which also support antibody class switching toward IgG4^13–15,24,25^. The positive correlation between Treg expansion and IgG4 observed in our cohort provides further support for the integrated pathway of humoral and cellular regulation. It was reported that AIT is associated with induction of both natural and inducible Treg subsets, including FoxP3⁺ Tregs, IL-10–secreting type 1 Tregs (Tr1), and regulatory follicular T cells, that collectively restrain allergy-promoting processes. In addition to direct suppression of effector T cells, these regulatory subsets influence dendritic cell maturation and function, skewing antigen presentation toward tolerogenic profiles and further reinforcing immune tolerance^15,26–29^.

On the other hand, the lack of significant change in total CD4⁺ T-cell percentages in the current study suggests that AIT selectively modulates regulatory subsets rather than inducing broad alterations in T-helper populations. This finding supports the utility of Treg quantification as a sensitive cellular biomarker for immune tolerance assessment^30^.

While both SCIT and SLIT were effective in improving clinical outcomes, a complex immunological distinction was evident between the two routes. SCIT recipients exhibited higher IgG4 levels and a trend toward greater Treg expansion compared with SLIT, suggesting that the subcutaneous route may elicit more potent systemic immune modulation^31,32^. These findings are consistent with prior studies showing that SCIT delivers allergen directly to subcutaneous dendritic cells in deep lymphoid compartments, promoting robust T-cell tolerance and humoral isotype switching toward IgG4, whereas SLIT primarily engages mucosal dendritic cells and induces local tolerance with slightly weaker systemic responses^32–34^.

The superior IgG4 induction observed in SCIT may be attributed to more sustained and controlled allergen exposure, which enhances the recruitment of allergen-specific B cells and promotes class switching to IgG4. In contrast, SLIT, while effective, may require higher or more prolonged dosing to achieve equivalent systemic IgG4 responses, consistent with previous observations in both pollen and HDM immunotherapy^35–37^. Treg expansion, although trending higher in SCIT, did not reach statistical significance, suggesting that both routes effectively induce peripheral tolerance, potentially through distinct cellular pathways. SLIT predominantly activates tolerogenic Langerhans cells in the oral mucosa, which migrate to local lymph nodes and promote Tr1 and FoxP3⁺ Treg induction, whereas SCIT engages systemic dendritic cells leading to broader effector T-cell suppression^38^.

Interestingly, despite these immunological differences, clinical outcomes assessed by ACT and RCAT scores were comparable between SCIT and SLIT recipients. This suggests that both routes ultimately converge on immune pathways sufficient to achieve symptomatic improvement, although SCIT may offer a more robust immunological signature^31–34^. These findings have practical implications: while SLIT may offer a safer and more convenient alternative for patients, SCIT may be preferable when a stronger systemic immune modulation is desired, particularly in patients with severe or multi-sensitized disease^39,40^.

Moreover, beyond clinical symptom control, the observed improvement in ACT and RCAT scores may reflect a broader enhancement in patients’ quality of life. AIT has been consistently associated with improved physical, emotional, and social functioning in patients with allergic diseases^9^. Therefore, the clinical benefits demonstrated in this study likely extend beyond symptom reduction to meaningful improvements in daily living and patient well-being.

The correlation between IgG4, Tregs, and clinical improvement in both groups reinforces the concept that humoral and cellular biomarkers can serve as objective measures to monitor response and potentially guide personalized immunotherapy strategies. Early changes in IgG4 and Treg frequencies could help identify patients who are likely to benefit most from either SCIT or SLIT, enabling individualized treatment plans.

Although this study focused on short-term immunological changes over nine months, early increases in IgG4 and expansion of Tregs may have implications for long-term tolerance. A key novel aspect of our study is the concurrent evaluation of IgG4 and Treg dynamics in an Egyptian cohort with HDM allergy receiving AIT, a population underrepresented in immunotherapy research. Limitations of this study include the limited sample size and the single-center design, which may limit generalizability. Additionally, only peripheral blood was assessed; tissue-specific immune responses in the airway mucosa were not captured. Moreover, the classification of responders based on ≥ 20% improvement in ACT or RCAT scores was an operational definition used for exploratory purposes in this pilot cohort. Finally, a key limitation of this study is that only total IgG4 was measured, whereas allergen-specific IgG4, which more precisely reflects immunotherapy-induced immune tolerance, was not assessed. Therefore, the observed increase in total IgG4 may not fully represent allergen-specific immunological responses.

Future studies with larger sample sizes should be performed to detect allergen-specific IgG4. Also, longer follow-up is necessary to determine whether the immunological differences observed between SCIT and SLIT translate into long-term clinical advantage or sustained tolerance.

## Conclusion

In conclusion, AIT improves clinical outcomes in adults with HDM allergy and is associated with increased IgG4 levels and expansion of regulatory T cells. SCIT produced stronger immunological responses than SLIT, although both treatments resulted in similar symptom improvement. Patients who responded to therapy showed higher IgG4 and Treg levels and lower IgE levels than non-responders, suggesting that these markers may help predict treatment success and support personalized immunotherapy approaches.

## Data Availability

All the data needed to support the current findings will be available upon request.
